# The *Arabidopsis thaliana* F-Box Protein FBL17 Is Essential for Progression through the Second Mitosis during Pollen Development

**DOI:** 10.1371/journal.pone.0004780

**Published:** 2009-03-11

**Authors:** Andi Gusti, Nicolas Baumberger, Moritz Nowack, Stefan Pusch, Herfried Eisler, Thomas Potuschak, Lieven De Veylder, Arp Schnittger, Pascal Genschik

**Affiliations:** 1 Institut de Biologie Moléculaire des Plantes du CNRS, Université de Strasbourg, Strasbourg, France; 2 Unigruppe am Max-Planck-Institut für Züchtungsforschung, Max-Delbrück-Laboratorium, Lehrstuhl für Botanik III, Universität Köln, Köln, Germany; 3 Department of Plant Systems Biology, Flanders Institute for Biotechnology (VIB), Gent, Belgium; 4 Department of Molecular Genetics, Ghent University, Gent, Belgium; Umeå Plant Science Centre, Sweden

## Abstract

In fungi and metazoans, the SCF-type Ubiquitin protein ligases (E3s) play a critical role in cell cycle regulation by degrading negative regulators, such as cell cycle-dependent kinase inhibitors (CKIs) at the G1-to-S-phase checkpoint. Here we report that FBL17, an *Arabidopsis thaliana* F-box protein, is involved in cell cycle regulation during male gametogenesis. *FBL17* expression is strongly enhanced in plants co-expressing E2Fa and DPa, transcription factors that promote S-phase entry. *FBL17* loss-of-function mutants fail to undergo pollen mitosis II, which generates the two sperm cells in mature *A. thaliana* pollen. Nonetheless, the single sperm cell-like cell in *fbl17* mutants is functional but will exclusively fertilize the egg cell of the female gametophyte, giving rise to an embryo that will later abort, most likely due to the lack of functional endosperm. Seed abortion can, however, be overcome by mutations in *FIE*, a component of the Polycomb group complex, overall resembling loss-of-function mutations in the *A. thaliana* cyclin-dependent kinase CDKA;1. Finally we identified ASK11, as an SKP1-like partner protein of FBL17 and discuss a possible mechanism how SCF^FBL17^ may regulate cell division during male gametogenesis.

## Introduction

Regulation of protein stability through the ubiquitin proteasome system (UPS) is a major mechanism underlying many different cellular and organismal processes, such as cell division, DNA repair, quality control of newly produced proteins, regulation of developmental pathways, important parts of immune defence and in plants, light and phytohormone signal transduction [1]–[3]. Degradation *via* the UPS is a two-step process: the target protein is first tagged by covalent attachment of ubiquitin and subsequently degraded by a multicatalytic protease complex called the 26S proteasome. Conjugation of ubiquitin to the protein involves a cascade of three enzymes: E1, E2 and E3. Ubiquitin-activating enzyme (E1) forms a high-energy thioester intermediate, E1-S∼Ubi, which is then trans-esterified to one of the several ubiquitin conjugating enzymes (E2s). The transfer of ubiquitin from the E2-S∼Ubi to an ε-NH2 group of an internal lysine residue in the target protein, requires an ubiquitin protein-ligase (E3). Because E3 enzymes determine the substrate specificity, they are recognized to play the most important role in the ubiquitylation reaction.

Among the different classes of E3s, two of them, the SCF and the Anaphase Promoting Complex/Cyclosome (APC/C), dominate cell cycle regulation, such as DNA replication and cell division, respectively. Whereas the APC/C permits progression and exit from mitosis by inducing proteolysis of different cell cycle regulators including PDS1/SECURIN and CYCLIN B (reviewed in [4]), the budding yeast SCF^CDC4^ and the mammalian SCF^SKP2^ (the name of the F-box protein being indicated in uppercase) are required to destroy the cell cycle-dependent kinase inhibitors (CKIs) SIC1 and p27^Kip1^, respectively (reviewed in [5], [6]) and thus promote the entry into S-phase. It is noteworthy that the human SCF^SKP2^ E3 targets other essential regulators of S-phase progression, including cyclin E [7], E2F1 [8], the RB-like p130 protein [9] and the licensing factor for DNA replication CDT1 [10].

In plants, the role of the APC/C as a mitotic regulator has been established (discussed in [11]), however the proteolytic machinery at the G1/S-phase transition is still poorly characterized. Nevertheless, in the model plant *A. thaliana*, loss-of-function mutants in *CULLIN1* arrest embryogenesis very early at the zygote stage [12], which is consistent with a role of an SCF E3 in cell cycle control. Moreover, two F-box proteins, similar to the metazoan SKP2, called SKP2A and SKP2B, have been identified in *A. thaliana* [13]–[15]. SCF^SKP2A^ recruits cyclin-dependent kinase (CDK)-phosphorylated E2Fc, a negative regulator of cell division and its partner DPb, for proteolysis [13], [14]. In addition, plant genomes encode also proteins sharing a short amino acid motif with the mammalian KIP/CIP-type CKIs [16]–[18]. *A. thaliana* counts seven such proteins called ICK1/KRP1, ICK2/KRP2, KRP3, KRP4, KRP5, KRP6 and KRP7. Despite poor homology conservation with the metazoan CIP/KIP proteins, plant ICK/KRPs bind to and inhibit several CDK complexes (likely A-type CDK associated to A- or D-type cyclins) (reviewed in [19]). At least two *A. thaliana* ICK/KRPs, ICK1/KRP1 and ICK2/KRP2, are degraded by the 26S proteasome [20], [21]. SCF^SKP2B^ may participate in the degradation of *A. thaliana* ICK/KRPs, because overexpression of the F-box protein SKP2B decreases the accumulation of ectopically expressed ICK/KRP1 and suppressed the ICK1/KRP1-dependent serrated leaf phenotype [15]. However, *skp2a skp2b* double mutant plants develop normally and do not stabilize ICK1/KRP1 [15], indicating a residual but sufficient activity of SCF^SKP2^ in this *skp2* mutant background and/or multiple E3s targeting redundantly ICK/KRPs.

Here, we characterized a novel F-box protein from *A. thaliana* called FBL17 that is involved in cell cycle regulation during pollen development. Reproduction in angiosperms relies on the production of two types of spores, microspores and megaspores, that give rise to male and female gametophytes, respectively [22], [23]. After meiosis, each microspore is first subjected to an asymmetric cell division (pollen mitosis I, PMI) producing the vegetative cell and a generative cell. Whereas cell division is arrested in the vegetative cell, the generative cell undergoes a second cell division (pollen mitosis II, PMII) leading to two sperm cells.

Loss-of function of *FBL17* impairs PMII resulting in bicellular pollen. The single sperm cell, however, can fertilize the egg cell of the female gametophyte, giving rise to an embryo that will abort, most likely by lack of functional endosperm. This phenotype is similar to the loss-of-function of the central cell cycle regulator cyclin-dependent kinase A;1 (CDKA;1) [24], [25]. A model explaining how SCF^FBL17^ could regulate PMII will be discussed.

## Results

### *FBL17* is an essential gene in *Arabidopsis*

**Figure 1:**
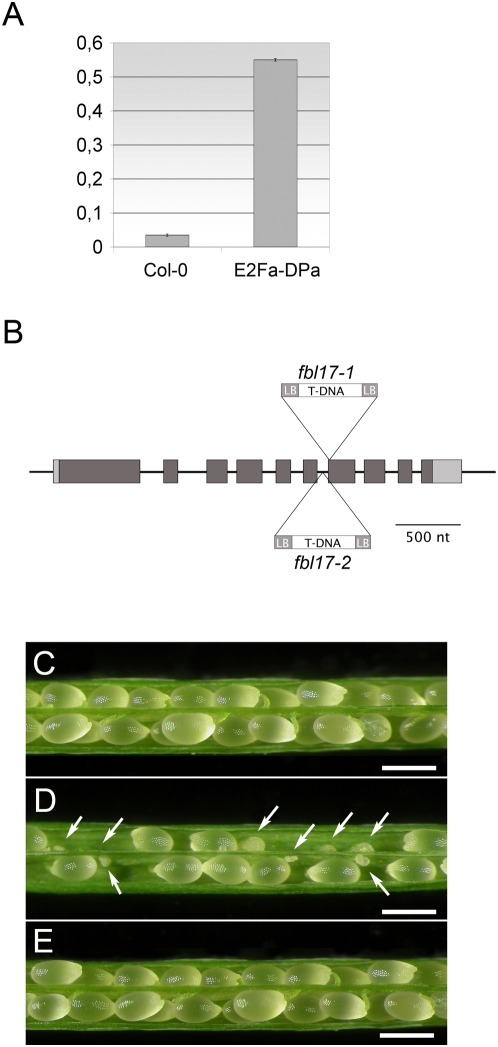
*fbl17* T-DNA insertion mutants. (A) FBL17 transcript accumulation in plants over-expressing the E2Fa transcription factor and its dimerization partner, DPa. Quantitative RT-PCR on RNA extracted from E2Fa-DPa overexpressing (OE) seedlings show a 15-fold increase in the relative abundance of *FBL17* transcript compared to control RNA (Col-0). The experiment was three times repeated. Data are means±SE. (B) Diagram of the genomic locus of FBL17. The two T-DNA insertions disrupt the 7^th^ exon and the 6^th^ intron in the *fbl17-1* and *fbl17-2* allele respectively. Light grey filling indicate non-translated region of the transcript whereas dark grey filling indicates coding sequence. (C) Wild type silique opened to reveal the seed content. (D) Heterozygous *fbl17-1^+/−^* silique displaying a reduced fertility and aborted seeds (marked by white arrows). (E) Homozygous *fbl17-1* mutant complemented with the *FBL17* genomic clone show wild type siliques and normal seed development. (C, D, E, bar = 500 µm).

To better characterize the role of the SCF in plant cell cycle function, we searched for F-box protein encoding genes that are expressed in a cell cycle dependent manner. Among the ∼700 *A. thaliana* F-box proteins [26], one of them, called FBL17 (At3g54650) was of particular interest because its expression was found to be 2.5 fold increased in S-phase based on available microarray data [27]. Moreover, this gene was *in silico* identified as a putative target of E2F transcription factors [28]. *A. thaliana* plants co-expressing ectopically E2Fa with its dimerization partner, DPa, display strongly induced cell proliferation rates [29] and indeed in these plants, we found a 15-fold increase in *FBL17* transcript accumulation (Figure 1A).

FBL17 is an LRR-type F-box protein, which has no close paralog in *Arabidopsis*. To gain insights into the function of *FBL17*, we isolated two *A. thaliana* T-DNA insertion mutants, named *fbl17-1* and *fbl17-2*, in which the T-DNA insertions interrupt the 7^th^ exon and 6^th^ intron, respectively (Figure 1B). Ninety-six *fbl17-1* and 102 *fbl17-2* seedlings were genotyped and none of them was found homozygous for the mutation, suggesting that *FBL17* is an essential gene in *Arabidopsis*. However, heterozygous *fbl17-1* and *fbl17-2* plants appeared normal in morphology during sporophytic development. As both lines contained single T-DNA insertions with integral sulphadiazine selection markers, we self-pollinated heterozygous mutant plants and analyzed the segregation of this marker in their progeny. This genetic analysis revealed a segregation ratio close to 1∶1 (53% of Sulf^R^ plants at most; Table 1), which is consistent with a gametophytic defect. Moreover, open siliques from self-pollinated *fbl17-1* and *fbl17-2* heterozygous plants showed a large proportion of aborted seeds (∼44%, see Table 2 and Figure 1C–E). Therefore we analyzed the T-DNA transmission of *fbl17-1* and *fbl17-2* by reciprocal crosses with wild type plants. A severe reduction in the transmission rate was only observed when heterozygous *fbl17* mutants were used as a paternal partner (Table 1).

**Table 1:** Genetic analysis of *fbl17* mutant plants.

Parental genotype (female×male)	F1 plants genotype (Sulfadiazine resistance)		n	*fbl17(%)*	TE (%)
	Sulf^R^	Sulf^s^			
*fbl17-1* (selfed)	171	193	364	47.0	NA
*fbl17-2* (selfed)	111	99	210	52.9	NA
Col-0×*fbl17-1*	17	293	309	5.5	5.8
*fbl17-1*×Col-0	187	191	378	49.5	97.9
Col-0×*fbl17-2*	23	573	596	3.9	4.0
*fbl17-2*×Col-0	316	348	664	47.6	90.8

Resistance to sulfadiazine (Sulf^R^, sulfadiazine resistant seedlings; Sulf^S^, sulfadiazine sensitive seedlings) was used for the *AtFbl17-1* and *AtFbl17-2* plants. n = total number. Transmission efficiencies were calculated according to [59]: TE = Sulf^R^/Sulf^S^×100%.

**Table 2:** Seed abortion in *fbl17* mutants.

Parental genotype (female×male)	Normal (%)	Aborted (%)	Undeveloped (%)	n
*fbl17-1* (selfed)	53.00	44.85	2.15	466
*fbl17-2* (selfed)	56.00	43.70	0.29	341
Col-0×Col-0	96.35	3.99	0.26	384
*fbl17-1*×Col-0	97.10	1.76	0.53	567
*Col-0*×*fbl17-1*	60.35	39.33	0.32	623
*fbl17-1 (pFBL17∶FBL17)* (selfed)	96.80	2.83	0.35	283

n = total number.

To demonstrate that this phenotype is caused by the *fbl17-1* mutation, we engineered a rescue construct (*pFBL17∶FBL17*) consisting of 868-bp of the promoter sequence fused to *FBL17* full length genomic sequence. Transgenic lines expressing this construction fully complemented the *fbl17-1* seed abortion phenotype (Table 2, Figure 1C–E).

### *fbl17* mutants produce pollen with only a single sperm cell

**Figure 2:**
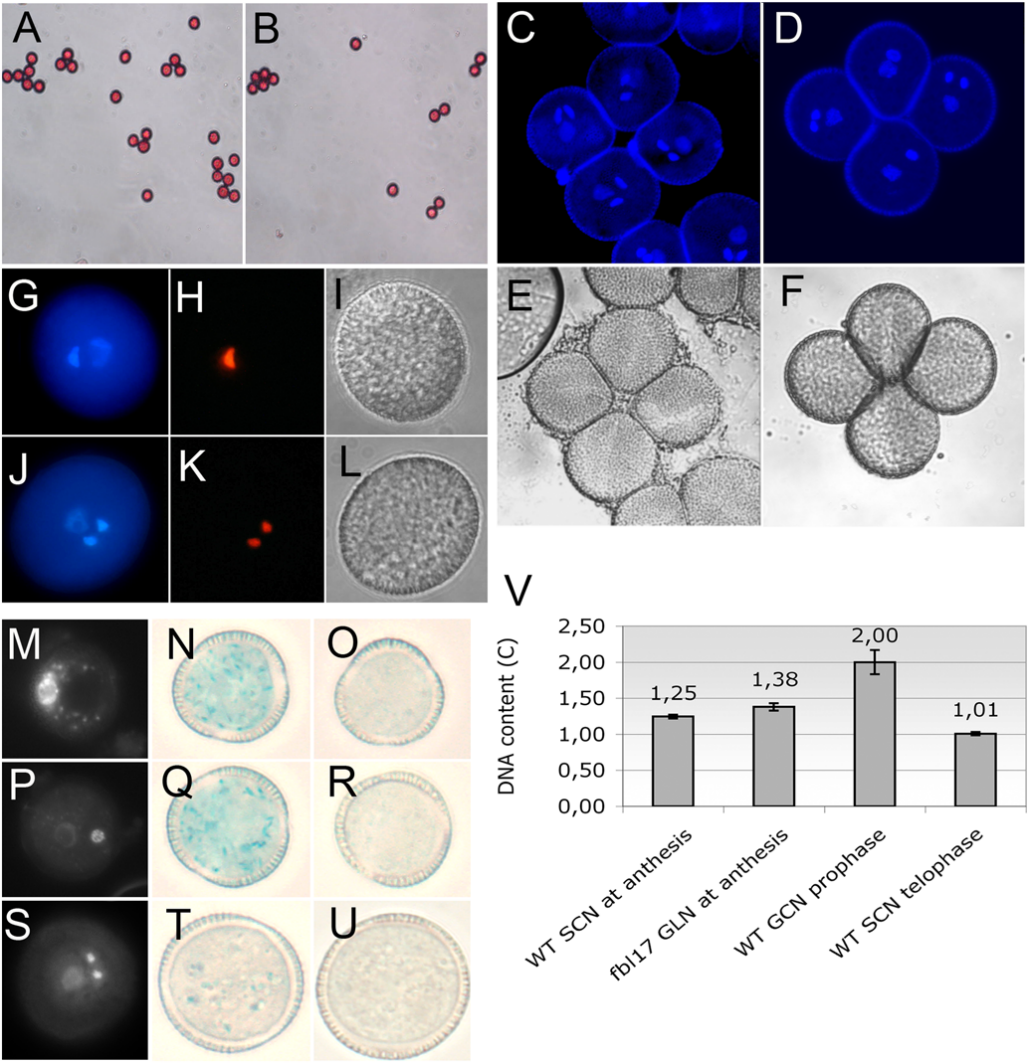
Pollen phenotype of *fbl17* mutants and *FBL17* expression during male gametogenesis. *fbl17* (A) and wild type (B) mature pollen viability test by coloration with Alexander's stain. The purple staining indicates that the grains are viable. Dehiscent pollen of *qrt1-1^−/−^* (C) and *qrt1-1^−/−^*, *fbl17-1^+/−^* (D) stained with DAPI and observed under UV fluorescence. The four pollen grains of the tetrad show two densely stained sperm cell nuclei and one large diffuse vegetative cell nuclei in *qrt-1* mutants, whereas two pollen grains of the tetrad show only a single germ cell nuclei in *qrt1-1,fbl17-1^+/−^* double mutants. (E, F) transmitted light picture of C and D. (G–L) Expression of the *HT10* gene in the *fbl17* mutant pollen. Expression of the HTR10-mRFPprotein under the HTR10 promoter in *fbl17-1* (H) and wild type (K) pollen, counterstained with DAPI (G, *fbl17-1*; J, wild type). (I, L) transmitted light pictures of G and J. (A, B) bar = 100 µm; (C–L) bar = 10 µm. (M–U) Promoter-GUS analysis of *FBL17* expression in pollen. (M, P, S) DAPI staining is applied to reveal the developmental stage of the pollen grain. (N–U) X-Gluc histochemical staining of pFBL17∶GUS (N, Q, F) and non-transformed Col-0 (O, R, U) pollen grains. Bars = 10 µm (V) DNA content measurement of wild type germinative cell nuclei at prophase (n = 9; DNA = 2C), wild type sperm cell nuclei at telophase (n = 16), WT sperm cell nuclei at anthesis (n = 111) in comparison to the unique germ-cell like nuclei of *fbl17* pollen (n = 77). Error bars = standard error mean.

A failure to transmit mutant alleles through the pollen can be caused by defects in pollen viability and/or development, germination, pollen tube growth or even fertilization. To test for pollen viability, we used Alexander's staining [30]. Similarly to mature pollen grains from wild type plants, pollen from heterozygous *fbl17-1* and *fbl17-2* mutants appeared full, round and red-stained when treated with Alexander's stain (Figure 2A,B), showing that the mutation does not affect pollen viability.

Next, we investigated pollen development. In *A. thaliana* plants, after meiosis, wild type haploid microspores undergo a first mitotic division resulting in a vegetative cell and a generative cell, which divides a second time. Thus, wild type mature pollen contains one large vegetative cell and two sperm cells. Strikingly, about 45% of the heterozygous *fbl17-1* and *fbl17-2* mature mutant pollen exhibited only one generative-like cell (Table 3), indicating a failure to progress through the second mitotic division. To analyze this in more detail, we performed a tetrad analysis by crossing *fbl17-1* to quartet mutation *qrt1-1* [31], which causes the four products of male meiosis to remain attached (Figure 2C–F). Pollen produced by *flb17-1^+/−^*; *qrt1-1^−/−^* plants never contained more than two aberrant grains and rarely contained fewer, indicating that *fbl17-1* is a highly penetrant male-gametophytic mutation.

**Table 3:** Phenotype of *fbl17* pollen at anther dehiscence.

	Normal (%)	Abnormal (%)	n
Col-0	97	3	1,355
*fbl17-1*	56	44	1,483
*fbl17-2*	55	45	1,492

Pollen from dehiscent anthers of WT and fbl17 ^+/−^ plants was stained with DAPI and observed under UV illumination. n = total number. Normal pollen all showed two sperm cells. Abnormal pollen showed a single sperm-like cell.

To confirm that *FBL17* is expressed in mature pollen as suggested by microarray data [32], we generated a GUS promoter-reporter with the *FBL17* promoter sequence that we have above shown to be functional, and introduced it into wild type plants. Histochemical staining revealed that *FBL17* expression is detectable during the microspore to bi-cellular pollen transition and fades later when pollen reaches the tricellular stage (Figure 2M–U).

To examine whether *fbl17-1* mutation affects pollen cell fate, we introduced in the *fbl17^+/−^* background the H3.3 histone variant pHTR10-HTR10-mRFP marker, which is specifically expressed in the male germ line and accumulates in the nuclei of wild type generative cell and subsequently in sperm cells [33]. Expression of this marker was observed in the generative-like cell nuclei of *fbl17-1* mutant (n = 95), indicating that this cell has not lost its gametic fate (Figure 2G–L). Furthermore, the single generative-like cell nuclei of *fbl17* mutant pollen is distinctively larger and less dense than the sperm cell nuclei observed in wild type pollen at anthesis (Figure 2D) and its morphology is reminiscent of the generative cell nuclei before the second mitotic division. Accordingly, measurement of DNA content by densitometry of DAPI stained pollen nuclei revealed that *fbl17* single generative-like cell nuclei have a slightly higher content than the wild type sperm cell nuclei at anthesis (1,38C vs 1,25C respectively; Figure 2V). Thus, the *fbl17* generative-like cell fails to progress into mitosis but has initiated S-phase reminiscent of wild-type sperms before fertilization. This suggested that the single generative-like cell of *fbl17* mutant pollen might function as a sperm cell.

### *fbl17* single sperm cell predominantly fertilizes the egg cell in the embryo sac and causes seed abortion

Overall, our results indicate that *fbl17* mutation prevents entry of the generative cell into mitosis, supporting a function of the FBL17 protein in cell cycle regulation in the male germ line. Interestingly, the production of a single sperm cell has recently been described for the loss of function of CDKA;1, the central *A. thaliana* cyclin-dependent kinase [24], [25]. In these studies, it was found that the single sperm cell of *cdka;1* may fertilize preferentially the egg cell in the embryo sac, leading to embryo arrest at the globular stage and fertilization-independent endosperm development.

**Figure 3:**
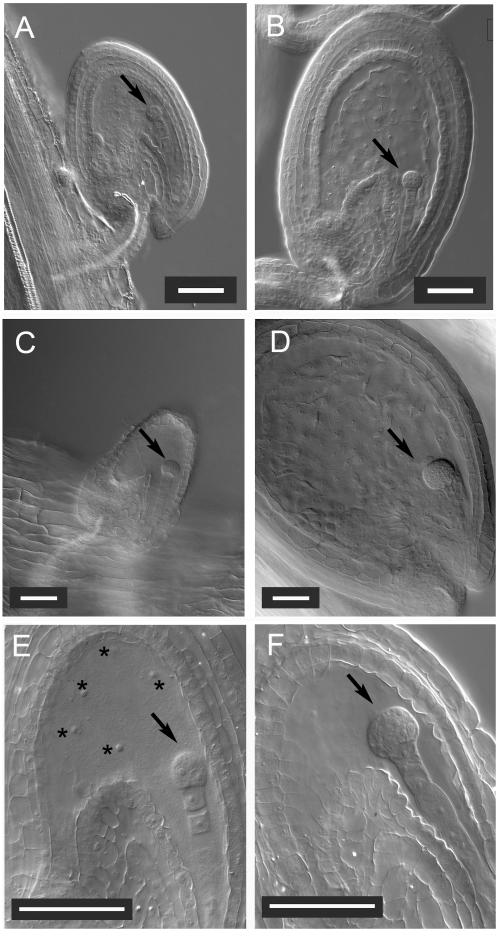
Fertilization with *fbl17* mutant pollen leads to embryo developmental arrest and seed abortion. Embryo development in wild type plants fertilized with *fbl17-1* mutant (A, E) and wild type (B) pollen, 3 days after pollination. Embryo development in wild type plants fertilized with *fbl17-1* mutant (C, F) and wild type (D) pollen, 4 days after pollination. Early fertilization-independent development of the endosperm is visible in *fbl17-1*-fertilized seeds (E) as evidenced by the presence of multiple nuclei (asterisks). Arrested globular embryo in *fbl17-1* mutant seeds with degenerated endosperm (F). Bars: 50 µm.

To further investigate the functionality of the single generative-like cell and the origin of semi-sterility of *fbl17* plants, we characterized the embryo sac development of plants fertilized with *fbl17* pollen. Siliques of selfed *fbl17-1* and *fbl17-2* plants showed arrested embryos at the globular stage with underdeveloped endosperm (Figure 3). These embryos never developed further and eventually degenerated. When *fbl17-*1 pollen was used to fertilize wild type plants, the same phenotype was observed (Figure 3A, C, E, F). Hence, *fbl17* mutants fully phenocopy the *cdka;1* mutation.

### *fbl17* embryos are rescued by fis-class mutants

Malfunction of the FIS-Polycomb group complex leads to central cell proliferation even in the absence of fertilization (reviewed in [34]–[36]). A striking observation was that mutations of genes encoding this complex, including *mea*, *fis2* and *fie*, could rescue the *cdka;1* paternally conferred seed abortion phenotype and allowed the formation of viable seeds with only a diploid endosperm [37]. To test whether *flb17* fertilized ovules behave in a similar manner, we crossed female *fie^+/−^* mutant with pollen from the heterozygous *fbl17-1* and *fbl17-2* mutants. The F1 generation was then genotyped to assess the transmission of both the *fbl17* and *fie* mutant alleles (Table 4). In contrast with Col-0 fertilized by *fbl17* mutants that only allowed 6–8% of transmission efficiency of the *fbl17* mutant allele, the *fie* mutation raised the transmission efficiency of *fbl17* to 27–32%. At the same time, the transmission efficiency of the *fie* mutant allele through the female gametophyte increased from 8% in crosses with wild-type pollen to 28–32% indicating a mutual rescue of *fbl17* and *fie* similar to what has been previously reported for *fis* mutants and *cdka;1*. Taken together, we conclude that the single *fb17* generative-like cell is able to some degree to function as a sperm cell leading to the production of viable seed when sufficiently developed endosperm is provided.

**Table 4:** Transmission frequencies of f*bl17* alleles in a fis-class mutant.

Parental genotype (female×male)	Genotype of viable progeny (%)			*TE% fbl17*	*TE% fie*	*Association factor*	n
	*fbl17*	*fie*	*fbl17/fie*				
Col-0×*fbl17-1*	6.4	NA	NA	6.5	NA	NA	294
Col-0×*fbl17-2*	8.5	NA	NA	8.4	NA	NA	142
*fie*×Col-0	NA	4.8	NA	NA	8.0	NA	248
*fie×fbl17-1*	32.3	31.8	27.7	32.3	31.8	87.1	393
*fie×fbl17-2*	30	28.1	23.7	26.9	28.1	84.5	438

N, number of F1 germinated seedlings scored; NA, not applicable; association factor, number of F1 plants carrying both a fis-class mutant (fie) and a *fbl17* mutant allele divided by the total number of plants carrying a *fis*-class mutant allele expressed in per cent.

### FBL17 interacts with specific ASKs to form SCF-like complexes

**Figure 4:**
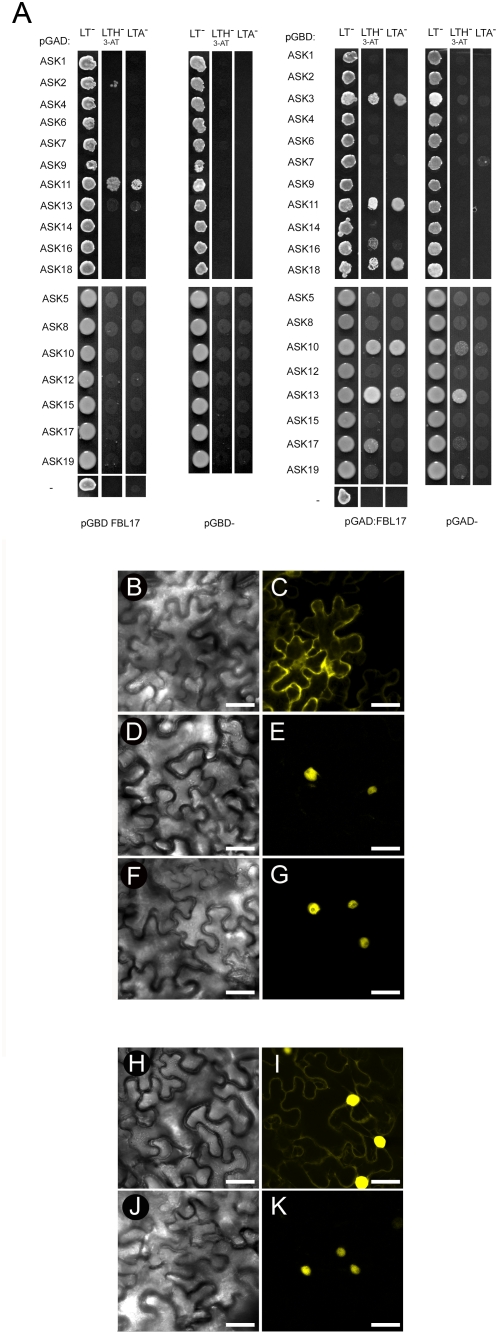
FBL17 interacts with a subset of ASKs. (A) Yeast-two-hybrid analysis of the interaction between FBL17 and ASKs. Yeast were grown for 3 days at 28°C. LTH- 3AT, low stringency selection, LTA, high stringency selection. Negative controls were done with empty bait vectors (pGBD) or empty prey vectors (pGAD). (B–G) Subcellular interaction of FBL17 with ASK11. Confocal laser-scanning micrographs of the abaxial surface of *N. benthamiana* leaves. (B, C) Transient expression of ASK11-YFP. The YFP signal is detected both in cytoplasm and nucleus. (D, E) Transient expression of FBL17-YFP. The signal is exclusively nuclear. (F–G) BiFC of FBL17-YN/ASK11-YC. Reconstitution of functional YFP as detected by YFP fluorescence occurs only in the nucleus. (H–K) Subcellular interaction of FBL17 with KRP7. (H, I) Transient expression of KRP7-YFP. A weak YFP signal in the cytoplasm and a strong signal in the nucleus can be detected. (J, K) BiFC of FBL17-YN/KRP7-YC. Reconstitution of functional YFP as detected by YFP fluorescence occurs only in the nucleus. (B, D, F, H, K, J) DIC images of the cells documented. (C, E, G, I, K) laser confocal micrograph of the YFP signal. Scale bars in B to K represent 45 µm.

Since FBL17 carries an F-box motif, we next examined whether this protein forms an SCF-type complex. Yeast two hybrid assays were conducted with the different *A. thaliana* SKP1-like proteins, called ASKs [38]. In yeast, several ASKs were able to interact with FBL17 when fused to the GAL4 DNA binding domain while failing to allow yeast growth on selective medium when fused to the activating domain (Figure 4A). In contrast, ASK11 displayed a robust interaction with FBL17 in both fusions. Interestingly, ASK11 is among the members of the *A. thaliana* ASK family that are strongly expressed in pollen [39], [40] and constitutes therefore a strong candidate for participating in an SCF^FBL17^ complex. It is noteworthy that the most predominant ASK, ASK1, was not found to interact with FBL17 in our yeast-two hybrid experiment. To further test a possible interaction between FBL17 and ASK11, we performed bimolecular fluorescence complementation (BiFC) experiments. As a control we fused to the N- and C-terminal half of YFP to the C-terminus of FBL17 and GUS, respectively. After co-injection of these fusion constructs into *Nicotiana benthamiana* leaf cells no YFP fluorescence could be found (data not shown). In contrast, when split-YFP fusions with FBL17 and ASK11 were co-injected, a strong fluorescence signal was recovered corroborating an interaction between these two components of an SCF-type complex in vivo (Figure 4B–G). Moreover transient expression of YFP fusions revealed that the FBL17 protein is essentially nuclear, whereas ASK11 localizes both to the cytoplasm and the nucleus (Figure 4B–E). Consequently, an interaction between ASK11 and FBL17 in the BiFC assays was restricted to nuclei (Figure 4F,G).

### FBL17 interacts with KRP7 in vivo

The FBL17 loss-of-function pollen phenotype is similar, if not identical, to the recently described *cdka;1* mutant [25] strongly supporting a cell cycle function of this F-box protein. An interesting hypothesis is that an SCF^FBL17^ E3 ligase targets one or more members of the plant cell cycle-dependent kinase inhibitors, called ICK/KRPs (reviewed in [19], [41]). In such a scenario, ICK/KRPs would accumulate in *fbl17* mutant pollen and consequently would inhibit the kinase activity mimicking the *cdka;1* mutant phenotype. To test this possibility, we first carried out yeast two-hybrid experiments, with binding and activation domain fusions of both FBL17 and all seven KRPs being tested. However, no strong interaction between FBL17 and KRPs were observed (not shown). This lack of protein interaction was, however, not unexpected because most SCF substrates require post-translational modifications [42], which may not occur in yeast. To circumvent this problem we performed bimolecular fluorescence complementation (BiFC) experiments with three ICK/KRPs, KRP3, KRP5 and KRP7 that are expressed in pollen [32]. We also used KRP1, which is not expressed in pollen, as a control. No signal could be recovered when split-YFP fusion constructs of KRP1, KRP3, or KRP5 were co-injected with FBL17 into *Nicotiana benthamiana* leaf cells (data not shown). In contrast, we observed a weak but distinct nuclear interaction when FBL17 and KRP7 were co-injected (Figure 4H–K).

KRP7 protein shows strong sequence similarity to KRP6, which was recently found to be expressed in pollen, as early as meiosis [43]. Interestingly, KRP6 turnover is necessary for cell cycle progression during gametogenesis. Thus we ordered *krp6* and *krp7* mutants from the stock centers but only *krp6* mutants could be obtained. In one T-DNA insertion mutant line, called *krp6-1*, KRP6 expression was abolished as indicated by qRT-PCR analysis (not shown). Homozygous *krp6-1* mutant plants showed no visible mutant phenotype. Strikingly, the *krp6-1^+/−^ fbl17-1^+/−^* double mutant, showed a partial rescue of *fbl17* pollen phenotype in F1 with the ratio of aberrant pollen of ∼35%, a number significantly lower than the ∼48% observed in *fbl17^+/−^* mutant (Table 5). From these experiments, we conclude that KRP6 and most likely KRP7 are targets of an SCF^FBL17^ during pollen development.

**Table 5:** Rescue of the *fbl17* pollen phenotype at anther dehiscence.

Genotype	Bicellular pollen (%)	Tricellular pollen (%)	n
Col-0	0	100	237
*fbl17-1*	48.1	51	235
*krp6*	0	100	255
*fbl17-1^+/−^ krp6-1^+/−^*	34.7	65.3	2,352

Pollen from dehiscent anthers of wild type and the indicated genotypes was stained with DAPI and observed under UV illumination. n = total number.

## Discussion

### FBL17 interacts with ASK11 and other ASKs

SCF-type complexes are involved in many developmental and physiological processes (reviewed in [44]). However so far, little is known about SCF complexes during gametophyte development. Here we investigated the function of FBL17, an F-box protein that is expressed during pollen development. Because FBL17 carries an N-terminal F-box domain, we first tested whether it associates with ASK proteins. The *A. thaliana* genome encodes 21 different ASKs [38]. However, the great majority of functionally characterized *A. thaliana* F-box proteins were shown to associate with ASK1 and ASK2, which exhibit broad expression patterns throughout plant development. Interestingly, ASK1 and ASK2 function during male meiosis [40] and in cell cycle regulation as cell division during embryogenesis is affected in the double *ask1 ask2* mutant [45]. Here we provide evidence that FBL17 can interact with several ASKs. In particular we observed the strongest interaction with ASK11, which is also expressed in pollen [39], [40] and known to interact with *A. thaliana* CUL1 [46]. Although we cannot exclude that FBL17 participates in many CUL1-type E3 complexes, our data supports the existence of a novel type of SCF E3 ligase, formed by CUL1, ASK11 and FBL17 regulating male germ line development.

### FBL17 is involved in cell cycle function during male gametogenesis

In fungi and metazoans, the SCF-types E3s play a critical role during the cell cycle at the G1-to-S-phase transition by degrading negative regulators such as CKIs. Whether such a mechanism is conserved in plants remained unclear. Consistently with this scenario, *A. thaliana cul1* loss-of-function mutants arrest early during embryogenesis at the zygote stage [12] and also exhibit a reduced transmission of the mutation through both male and female gametophytes. However, such a phenotype was not reported for any of the ∼700 F-box encoding genes in *A. thaliana*.

Here, we provide evidence that *FBL17* is a key regulator of the cell cycle during gametophyte development. First, *FBL17* is expressed at the G1-to-S-phase transition [27] and its expression is strongly enhanced in plants ectopically co-expressing the S-phase transcription factor E2Fa together with its dimerization partner DPa. It is noteworthy that FBL17 promoter sequence carries the canonical E2F binding sites in both *A. thaliana* and rice [28]. Whether *FBL17* is a direct target of E2Fs, however, is not established yet.

Second, the *FBL17* loss-of-function mutants arrest germ cell division leading to a single sperm cell phenotype that closely resembles (see also below) the phenotype found in mutants for the major cell cycle regulator; CDKA;1.

The paternal effect of *fbl17* mutants leads to seed abortion, which is characterised by an arrest of embryo development at the globular stage and a defect in endosperm development. Similarly to the *cdka;1* mutant [25], it is likely that the *fbl17* single sperm cell may preferentially fertilize the egg cell, but not the central cell in the embryo sac. In both cases, the uniparental underdeveloped endosperm, which is unable to nourish the embryo, may thus be the cause of seed abortion. Hence, when the arrest of endosperm development is released by the mutation of *FIE*, a component of the Polycomb group complex, we observed at least a partial rescue of the paternally conferred *fbl17* seed abortion phenotype.

### FBL17 targets negative regulator(s) of the cell cycle

Based on the pollen phenotype, we propose that FBL17 targets a negative regulator of the cell cycle. Because *fbl17* mutants phenocopy CDKA loss-of-function alleles, we speculated that the negative regulator targeted by FBL17 may directly act at the level of CDKA;1. Plausible candidate proteins are CKIs, whose accumulation during gametogenesis would lead to CDKA;1 inactivation and as a consequence cell cycle arrest. Indeed, we observed a physical interaction between FBL17 and at least one members of the KRP family (e.g., KRP7). Moreover, *KRP6* mutation suppressed, at least partially, the *fbl17* pollen phenotype. After the completion of this work Kim et al. [47] reported a similar role of *FBL17* during male gametogenesis. This study showed that both KRP6 and KRP7 are expressed in the male germ cells after asymmetric division, but disappear in sperm cells. Based on their in vitro degradation assays and the fact that a KRP6-GFP fusion protein remained stable in the *fbl17* single germ cell, these authors also proposed that KRP6 and KRP7 are the main target this SCF E3 ligase [47]. Nevertheless, because a *krp6 krp7* double loss-of-function mutant is not available, this model still awaits a definitive genetic prove.

An interesting question, which has not yet been fully answered, is at which stage the cell cycle *fbl17* mutant arrests? Based on our knowledge of the cell cycle in yeast and animals, a naïve prediction was that *fbl17* loss-of-function mutants would arrest at the G1/S transition. However, this was not the case because the mutant pollen had a higher DNA content than wild-type sperm and thus must have entered into S-phase ([47] and our work). In contrast, the DNA content of *fbl17* was measured to be lower than 2C and thus, the mutant sperms appear to be not arrested at the entry into mitosis. However, as the *fbl17* single germ cell fertilizes the egg cell, even allowing complete embryo development in a *fie* mutant background, we exclude that an aneuploid situation arose after fertilization and thus, it is likely that DNA replication was completed at the time of fertilization. Thus, we speculate that *fbl17* mutants are arrested in the G1 phase and similarly to wild type would be still susceptible to an S-phase trigger late in development. This trigger would then be able to overwrite the *FBL17* loss-of-function. It is noteworthy here that *FBL17* expression is abolished in late pollen developmental stages as based on our promoter-reporter lines and [47]. Alternatively, the loss of an SCF^FBL17^ complex might result in slow, but none the less steady progression into S-phase. This S-phase might then, similar to wild type, be only completed shortly before fertilization and thus, the generative-like-cells of *fbl17* mutants would resemble wild-type sperm cells.

### Multiple components of UPS regulate *A. thaliana* male gametogenesis

**Figure 5:**
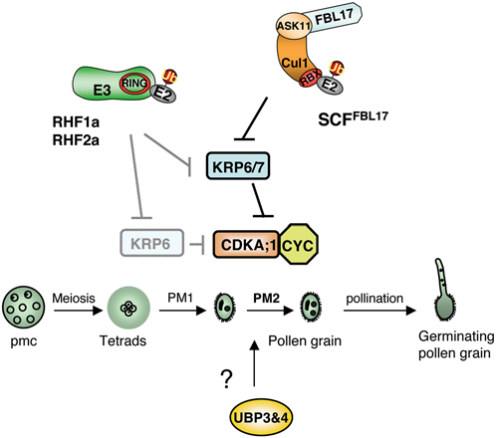
Model showing how the UPS could regulate the cell cycle during male gametogenesis. In this model RHF1a and RHF2a mediate KRP6 26S-proteasome-dependent degradation starting from meiosis [43]. *RHF1a* and *RHF2a* loss-of-function of leads to KRP6 accumulation and as a consequence blocks both PMI and PMII. The SCF^FBL17^ E3, most likely containing ASK11, mediates KRP6/7 (and eventually other proteins) is strictly required to allow PMII to occur ([47] and our work). The role of UBP3 and UBP4 during pollen development remains to be determined.

Recent work has shown that KRP6 is also targeted by two related RING-H2-type E3s, called RHF1a and RHF2a, during pollen development [43]. Contrarily to *fbl17*, the *rhf1a rhf2a* double mutant pollen phenotype is partially penetrant and pollen development is arrested at multiple stages. Constitutive overexpression of KRP6 could phenocopy the *rhf1a rhf2a* double mutant defects. Thus, ICK/KRP degradation is regulated at different levels during pollen development (Figure 5). Although additional experiments are required to determine the exact contribution of each E3 in KRP6/7 turnover, RHF1a and RHF2a do not seem to act redundantly with SCF^FBL17^ to allow entry into PMII.

With this, KRP turnover in plant cells, at least in the germ line, show similarities with the situation found in mammals where the cell cycle-dependent kinase inhibitor protein p27^KIP1^ is degraded by two different classes of E3s; the SCF^SKP2^ and the RING-type KPC1/KPC2 [48]. The KPC E3 mediates polyubiquitinylation of p27^KIP1^ in the cytoplasm in a phosphorylation-independent manner during G1 phase, whereas SCF^SKP2^ is involved in phosphorylated-dependent p27^KIP1^ degradation in the nucleus during S and G2 phases [49]. Whether SCF^FBL17^ recruits phosphorylated ICK/KRPs is unknown. Nevertheless, it has been shown that at least one *A. thaliana* ICK/KRP is subjected to proteolysis in a phosphorylation-dependent manner [21]. In agreement, putative CDKA;1 phosphorylation sites are present in both KRP6 and KRP7 protein sequences [41].

Not only ubiquitylation, but also deubiquitylation has recently been involved in pollen development [50]. Hence two closely related *A. thaliana* Ub-specific proteases (UBPs) called *UBP3* and *UBP4* are important for pollen mitosis II, as their combined disruption also leads to a single sperm cell, which however is unfertile. The target of UBP3/UBP4 is unknown. Interestingly, recent work in mammals identified USP19 as a UBP that regulates the protein level of KPC1, the catalytic subunit of KPC [51]. In their model, USP19 stabilizes KPC1 and thus indirectly promotes degradation of p27^KIP1^ in the cytoplasm. Whether the plant UBP3/UBP4 have a similar effect on ICK/KRP accumulation during pollen development is worth to investigate.

## Materials and Methods

### Plant material and growth conditions

*A. thaliana* plants used in this study were all derived from the Columbia-0 (Col-0) accession. The lines *qrt1-1* [31], *fbl17-1* (GABI-170E02) *fbl17-2* (GABI-436F11), *krp6* (SAIL_548-B03) and *fie* (GABI_362D08) were obtained from the NASC stock centre. The position of the T-DNA inserts in the *fbl17* lines were verified by DNA sequencing of the flanking regions amplified with primer pairs GABI T-DNA LP/7, GABI T-DNA RP/8 for *fbl17-1* and GABI T-DNA LP/9, GABI T-DNA RP/10 for *fbl17-2* (see oligo table below the GABI web site: http://www.gabi-kat.de). Those primer pairs were also used for genotyping. The transgenic lines E2Fa/DPa over-expressing line and pHTR10-HTR10-mRFP have been described elsewhere [29], [33]. For most experiments, plants were grown on soil under standard greenhouse conditions (22°C, 16 h light photoperiod). Analysis of the *fbl17* alleles transmission efficiency and selection of heterozygous *fbl17* plants were performed by growing seedlings on sterile 1× Murashige and Skoog agar medium supplemented with 10 µg/ml sulfadiazine.

### Constructs and transformation

The complementation genomic construct was generated by insertion of a 3747nt-long PCR amplified genomic product from (–)868 bp to the stop codon into the *Not*I site of pCBI [52]. PCR amplification of this genomic fragment was done with the primers 11 and 12 (see table below). The construct was transformed into *fbl17-1* mutant using *Agrobacterium tumefaciens* GV31O1 with the floral-dip methods [53]. Primers 3 and 4 were used to identify homozygous knock-out mutants rescued by the full genomic construct. The *promFBL17∶GUS* construct was created by PCR amplifying 868nt of the promoter region with primers 5 and 6 and cloning of the fragment in pENTR1A vector between the *EcoR*I and *Xho*I sites. The promoter fragment was then remobilized by LR clonase II recombination (Invitrogen) into pMDC162 [54]. For the BiFc constructs *FBL17*, *ASK11*, *KRP1*, *3*, *5* and *7* cDNAs were first amplified by PCR without their STOP codons, cloned into pDONR 201 (except *FBL17* which was cloned into pDONR 207) by BP clonase recombination (Invitrogen) before to be transferred into the split-YFP destination vector pSYN and pSYC [20] by LR clonase reaction (Invitrogen). 35S∶YFP fusion were obtained by remobilizing *FBL17*, *ASK11*, and *KRPs* cDNA from the pDONR vector to the plasmid pEXSG-YFP [55].

### Primers

***Oligo*** **Sequence (5′→3′)**

**1** TCGAGAGTGATTTTGACGCGACG

**2** CCGAGAGCCAAAGAGTGGAGAG

**3** GTAAATTCTTGATCTTTGGTTTGCA

**4** GTTTTTCCATTTTGTAAGATATTTG

**5** AAAAAGCAGGCTCGTGAGATTTTGGGAG

**6** AGAAAGCTGGGTATCACCAAATCCTTGAG

**7** GTCAGTTTCCTTTTTATCCAG

**8** GACGAAAATTGTGACGAGTCC

**9** GCCGAGAAGTTTTCAGAAACC

**10** TGTCAGTTTCCTTTTTATCCAGG

**11** ATAAGAATGCGGCCGCTGTATATGATTTGCGAG

**12** ATAAGAATGCGGCCGCGATGAACAAGATTAGAG

**13** gaaaccgaaaccgaaacctc

**14** ccctcactcactggactcgt

### Q-PCR

Total RNA for Q-PCR was extracted from 6 days-old E2Fa-DPa ^OE^ and Col-0 wild-type seedlings grown on sterile 1× Murashige and Skoog agar medium with the Plant RNeasy kit (Qiagen). For *krp6-1* mutant analysis, total RNA was extracted from inflorescences of *krp6-1* homozygous mutant and Col-0 wild-type plants. 1 µg of total RNA was reverse transcribed with the Superscript II RT kit (Invitrogen) and a mixture of random hexamers and oligo dT (18) according to the manufacturer's instruction. PCR was performed using oligos 1 and 2 (*FBL17* specific) and oligos 13 and 14 (*KRP6* specific) in a total volume of 15 µL SYBR Green Master mix (Roche) on a Lightcycler LC480 apparatus (Roche) according to the manufacturer's instructions. The *ACTIN2*, *AT4G34270* and *AT4G26410* genes were used as internal controls for the normalization of the q-PCR.

### Microscopy

Developing seeds were prepared from silliques at different days after pollination (DAP) and mounted on microscope slides in a clearing solution of 8∶2∶1 Chloral hydrate: distilled water: glycerol as described in [56]. Observations were performed on a Zeiss Axiophot using 20× and 40× DIC optic. Pollen viability was assessed by mounting pollen in Alexander's stain [30] and observed by transmitted light microscopy. For DAPI staining of pollen grains, anthers at different stage were dissected and mounted in DAPI solution (DAPI 5 µg/ml, PIPES 50 mM, EGTA 5 mM, NP-40 0,1%, and DMSO 10%) and incubated for 30′ before the observation on a Nikon fluorescent stereo-microscope E800 equipped with a 100× optic. Quantification of DNA content was performed with the software ImageJ (http://rsbweb.nih.gov/ij/) on images taken with constant settings. Background fluorescence values were subtracted to the measure to correct for difference in staining intensity. The obtained measures were normalized against those of generative prophase nuclei from wild type bi-cellular pollen sampled in pre-dehiscent anthers, which are by definition at 2C. Pictures of *qrt-1/fbl17-1* and *qrt-1* mutant pollen were obtained on a Zeiss LSM 510 Meta confocal microscope as a Z-stack subsequently flattened into a single image in the ImageJ software. GUS staining of anthers at different stages was performed as described in [57] and the state of pollen development was determined from the pollen DAPI staining taken from the same flower at the same time.

### Yeast-two hybrid

The *ASK1* to *ASK19* cDNAs cloned as fusions to the GAL4 activation domain and GAL4 binding domain, respectively, in Gateway-compatible pGADT7 and pGBT9 (Clontech) yeast two-hybrid vector have been described in [46]. Full length FBL17 cDNA was mobilized from the entry vector pDONR FBL17 to both pGADT7 and pGBKT7 (Clonetech) with LR clonase II kit (Invitrogen). The yeast strain AH109 (*MATa*, *trp1-901*, *leu2-3*, *112*, *ura3-52*, *his3-200*, *gal4*, *gal80*, *LYS2::GAL1UAS-GAL1TATA-HIS3*, *GAL2UAS-GAL2TATA-ADE2*, *URA3::MEL1UAS-MEL1TATA-lacZ*, *MEL1*) was transformed with the appropriate combinations of bait and prey vectors. Transformants were selected on synthetic defined (SD)/-Leu/-Trp (SD- LW) medium for 2 days at 30°C. Weak and strong interactions were tested by transferring transformants on SD/-Leu/-Trp/-His (-LWH) with 5 mM 3-amino-1,2,4-triazole (3-AT) and SD/-Leu/-Trp/-Ade (-LWA) media, respectively, allowing growth for 3 days at 30°C.

### BiFc assay

For infiltration of *Nicotiana benthamiana* leaves the *A. tumefaciens* strain GV3101 pMP90RK was used. The *A. tumefaciens* strains containing the BiFc vectors were infiltrated as described [58]. Infiltration was performed on the abaxial leaf side of two-month-old plants and analyzed 3 to 5 days later.
